# Stringent Nucleotide Recognition by the Ribosome at the Middle Codon Position

**DOI:** 10.3390/molecules22091427

**Published:** 2017-08-29

**Authors:** Wei Liu, Dongwon Shin, Martin Ng, Karissa Y. Sanbonmatsu, Yitzhak Tor, Barry S. Cooperman

**Affiliations:** 1 Department of Chemistry, University of Pennsylvania, Philadelphia, PA 19104, USA; weiliu@sas.upenn.edu (W.L.); martinng@sas.upenn.edu (M.N.); 2 Department of Chemistry and Biochemistry, University of California, San Diego, La Jolla, CA 92093, USA; dshin@trilinkbiotech.com (D.S.); ytor@ucsd.edu (Y.T.); 3 Theoretical Biology and Biophysics Group, Los Alamos National Laboratory, Los Alamos, NM 87545, USA; kys@lanl.gov

**Keywords:** fluorescent mRNA, mRNA:tRNA base pairing, kinetic mechanism, steric constraint, atomic mutagenesis

## Abstract

Accurate translation of the genetic code depends on mRNA:tRNA codon:anticodon base pairing. Here we exploit an emissive, isosteric adenosine surrogate that allows direct measurement of the kinetics of codon:anticodon base formation during protein synthesis. Our results suggest that codon:anticodon base pairing is subject to tighter constraints at the middle position than at the 5′- and 3′-positions, and further suggest a sequential mechanism of formation of the three base pairs in the codon:anticodon helix.

## 1. Introduction

Great strides have been made in recent years toward understanding the complex process by which the ribosome utilizes tRNAs to mediate translation of the information encoded within mRNA as consecutive three-nucleotide codons into a polypeptide sequence, benefiting from results of structural, biochemical, biophysical and computational studies [1,2,3,4,5,6,7,8,9,10,11]. Detailed kinetic and dynamic studies have led to the formulation of quantitative schemes for the two major steps within the elongation cycle of peptide synthesis: (i) ternary complex (TC, consisting of aminoacyl(aa)-tRNA.EF-Tu.GTP) binding to a posttranslocation (POST) or 70S initiation complex (70SIC), followed by accommodation of aa-tRNA within the A-site of the ribosome and peptide bond formation, leading to pretranslocation (PRE) complex formation [5,9,10,12,13,14,15,16] and (ii) EF-G.GTP-catalyzed conversion of PRE to POST complex, by which tRNAs bound in the A- and P-sites are translocated, along with the mRNA codons to which they are bound, to the P- and E-sites, respectively [16,17,18,19,20,21,22,23]. These studies rely heavily on functional derivatives of tRNAs, ribosomal proteins, or translational factors, site-specifically labeled with either a fluorophore or a fluorescent quencher [4,16,20,21,22,23,24]. However, direct information about mRNA dynamics during elongation is quite limited, leaving many questions about the mechanism of codon:anticodon base pairing unanswered.

**Figure 1:**
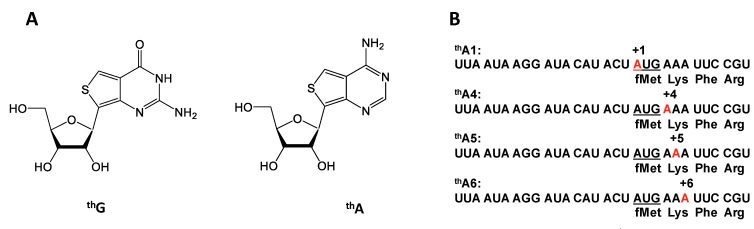
(**A**) Isosteric guanosine and adenosine surrogates ^th^G and ^th^A; (**B**) mRNAs site-specifically labeled with ^th^A (in red).

Emissive and responsive isosteric mimetics for purine nucleosides, denoted ^th^G and ^th^A (Figure 1A), which can participate in normal Watson-Crick base pairing, have recently been described [25,26,27]. Previously we demonstrated that changes in the fluorescence of mRNAs containing ^th^G at each of the three positions within the first elongation codon [28] could be used to monitor rates of PRE and POST complex formation. Like ^th^G, ^th^A is strongly emissive (Ф = 0.21) (Figure S1) and its fluorescence is responsive to environmental changes [25]. In the present work we report experiments utilizing mRNAs encoding the tetrapeptide fMetLysPheArg, in which each of the adenosines in the first two codons, AUG and AAA encoding tRNA^fMet^ and tRNA^Lys^, respectively, is substituted, one at a time, by ^th^A, yielding labeled mRNAs denoted ^th^A1-mRNA, ^th^A4-mRNA, ^th^A5-mRNA, and ^th^A6-mRNA (Figure 1B). Our results allow comparison of the effects on both PRE and POST complex formation of substituting the more bulky, less polar, thiophene C-S-C grouping for the N^7^-C^8^-N^9^ purine grouping in adenosine, providing an example of using “atomic mutagenesis” [29,30] to elucidate structure:function relationships. Our results demonstrate the tight structural constraints to which A:U base pairing at the middle position is subject, and support a mechanism for the formation of the three base pairs in the codon:anticodon helix that proceeds sequentially rather than simultaneously.

## 2. Results

### 2.1. ^th^A Fluorescence Intensity Changes Accompanying PRE and POST Complex Formation

**Figure 2:**
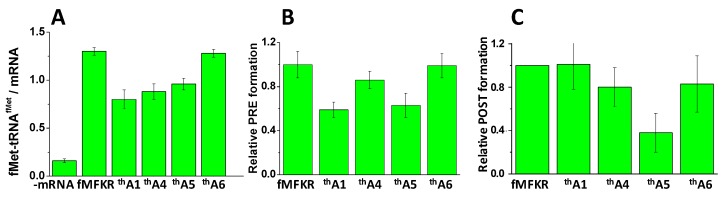
Activities of ^th^A-containing mRNAs in complex formation. (**A**) 70SIC formation, measured as fMet-tRNA^fMet^ co-sedimented per mRNA added: ribosomes, 1.0 mM; mRNA 0.5 mM; (**B**) Normalized PRE complex formation, with background in the absence of mRNA subtracted: ribosomes, 0.8 mM; (**C**) Normalized POST complex formation: ribosomes, 4.8 mM. Ranges shown are ± average deviation. Control experiments were performed, as indicated, with either no added mRNA, or with added unmodified mRNA. Values reported in (**B**) were normalized to the value obtained for unmodified fMKFR mRNA after subtraction of background obtained with no mRNA. Values reported in (**C**) were obtained following the previously published procedure for ^th^G5 [28] and normalized to the value obtained for unmodified fMKFR mRNA.

Ribosomes programmed with mRNAs ^th^A1, ^th^A4, ^th^A5, and ^th^A6 displayed substantial functionality in forming 70SIC (Figure 2A), PRE complex (Figure 2B), and POST complex (Figure 2C). Stoichiometries of complex formation, measured at a single standard condition, were generally ≥50% relative to ribosomes programmed with control mRNA lacking ^th^A, denoted fMKFR, although POST complex formation with ^th^A5 (30%) was clearly lower.

**Figure 3:**
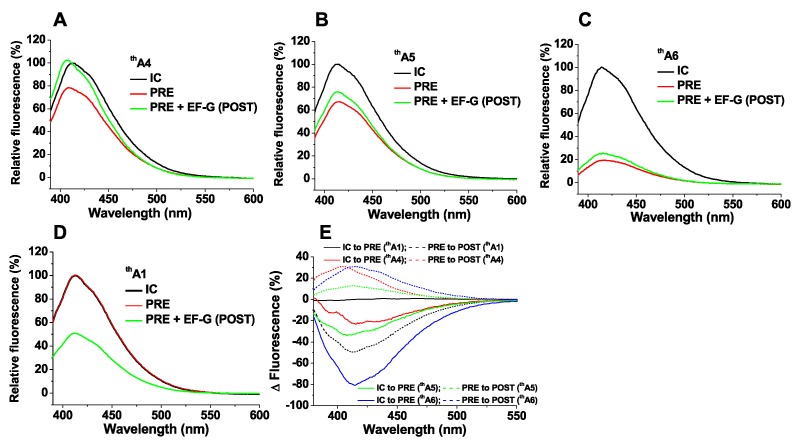
Fluorescence changes of ^th^A-containing mRNAs between ribosomal complex conversion (**A**–**D**); Traces are shown for purified 70SIC (black); PRE complex (red); POST complex (green); (**E**) Fluorescence intensity changes were obtained by subtracting the spectrum of a purified complex (70SIC or PRE) from the spectrum of a complex prepared in situ (PRE or POST, respectively) and then normalizing the intensity differences to the peak intensity value of purified complex (70SIC or PRE, respectively).

For 70SICs programmed with ^th^A4, ^th^A5 or ^th^A6, PRE complex formation on addition of cognate EF-Tu.GTP.Lys-tRNA^Lys^ (Lys-TC) leads, in each case, to significant decreases in fluorescence intensity (Figure 3A–C). These changes arise as a consequence of codon:anticodon interaction, since almost no changes are seen with ^th^A1 (Figure 3D) or on replacing Lys-TC with non-cognate Arg-TC (Figure S2A–C). Translocation of PRE complexes to POST complexes on addition of EF-G.GTP proceeds with a large decrease in the fluorescence intensity of ^th^A1 as tRNA^fMet^ dissociates from the ribosome (Figure 3D), but with moderate to negligible increases in the fluorescence intensities of ^th^A4, ^th^A5 and ^th^A6 (Figure 3A–C). These intensity changes, summarized in Figure 3E, provide spectroscopic signatures for monitoring the kinetics of PRE and POST complex formation during the polypeptide elongation cycle, as described below. Since ^th^A fluorescence responds to alterations in several factors, including solvation, base-pairing, stacking and interactions with proximal chromophores, it is not currently possible to ascribe fluorescence intensity changes to specific molecular perturbations in the ^th^A microenvironment.

### 2.2. Kinetics of PRE Complex Formation

**Figure 4:**
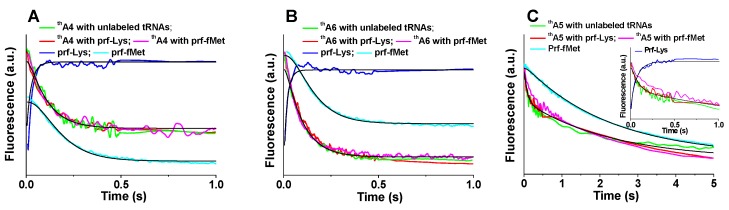
Kinetics of PRE complex formation. Ribosomes programmed with: (**A**) ^th^A4; (**B**) ^th^A6; (**C**) ^th^A5. Fluorescence changes on rapid mixing of 70SICs (0.1 μM) With Lys-tRNA^Lys^ (0.4 µM). Rates of ^th^A fluorescence change were virtually identical using either unlabeled fMet-tRNA^fMet^ and unlabeled aa-tRNA (green), unlabeled fMet-tRNA^fMet^ and Lys-tRNA^Lys^(prf) (red), or fMet-tRNA^fMet^(prf) and unlabeled Lys-tRNA^Lys^ (purple). Also shown are rates of fluorescence changes of Lys-tRNA^Lys^(prf) (dark blue) and of fMet-tRNA^fMet^(prf) (cyan). The inset in (**C**) shows an expanded time scale, making clear biphasic nature of ^th^A5 fluorescence change. Solid black lines are fitted to Scheme 1, using rate constants provided in Table 2.

We and others previously have used changes in the proflavin (prf) fluorescence intensities of aa-tRNA^aa^(prf) (as part of a TC) and fMet-tRNA^fMet^(prf) to monitor, respectively, the initial rates of TC binding to the 70SIC [12] and of aa-tRNA accommodation into the A-site prior to dipeptide formation [31]. In this work we compare the rates of prf fluorescence change of Lys-tRNA^Lys^(prf) and fMet-tRNA^fMet^(prf) with those obtained with ^th^A present in ^th^A4-mRNA (Figure 4A), ^th^A5-mRNA (Figure 4B), or ^th^A6-mRNA (Figure 4C) to determine the rates of different substeps within overall PRE complex formation. Reaction mixtures contained either a single fluorescent component, ^th^A alone, or two fluorescent components, ^th^A plus either Lys-tRNA^Lys^(prf) or fMet-tRNA^fMet^(prf). In the latter cases, changes in both ^th^A and prf fluorescence were monitored simultaneously.

Apparent rate constants, collected in Table 1, were evaluated using Equation (1) or Equation (2) (Section 4). In all three cases, the Lys-TC(prf) fluorescence change was the most rapid. The apparent first order constants for 70SICs programmed with ^th^A4-mRNA (Figure 4A), ^th^A6-mRNA (Figure 4C), and fMKFR-mRNA, 44–62 s^−1^ (Table 1), correspond closely to previously published values [12], whereas the value with ^th^A5-mRNA is ~3-fold slower (18 s^−1^) (Table 1). For both ^th^A4-mRNA and ^th^A6-mRNA, rates of fMet-tRNA^fMet^(prf) fluorescence change, which also agree with previously published values [31], are somewhat slower than those for ^th^A fluorescence change. These results lead to the conclusion that, for these labeled mRNAs, the change in ^th^A fluorescence that depends on codon:anticodon interaction follows TC binding and precedes Lys-tRNA^Lys^ accommodation, a kinetic mechanism that is consistent with the accepted view of the sequence of events involved in PRE complex formation [5,9].

**Scheme 1:**
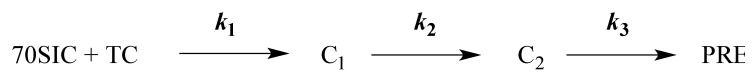
Kinetic scheme for ^th^A fluorescence changes.

This order of reaction is captured by globally fitting the results for ^th^A4-mRNA and ^th^A6-mRNA (Figure 4A,B) to Scheme 1 for PRE complex formation (more detailed schemes are presented in references [9] and [32]), in which steps 1, 2 and 3 correspond to initial binding of TC to 70SIC (Complex C_1_), formation of the codon:anticodon helix (Complex C_2_) and Lys-tRNA^Lys^ accommodation into the A-site followed by rapid peptide bond formation [5,12,32,33] to complete PRE complex formation, respectively. The fitted microscopic rate constants and relative fluorescence intensities for this Scheme are presented in Table 2.

The situation is more complicated for ^th^A5-mRNA (Figure 4C), for which the ^th^A fluorescence change has clear biphasic character, with a minor, fluorescence decrease that occurs *more* rapidly than the monophasic change seen with ^th^A4-mRNA or ^th^A6-mRNA and a major fluorescence change that occurs much less rapidly, at a rate that is quite similar to that observed for fMet-tRNA^fMet^(prf) fluorescence change (Table 1). These differences suggest that, although we are able to globally fit the results obtained with ^th^A5-mRNA to Scheme 1, giving the values for *k*_1_, *k*_2_, and *k*_3_ displayed in Table 2, the identity of C_2_ differs from that assigned in the case of ^th^A4-mRNA or ^th^A6-mRNA (see Section 3).

### 2.3. Kinetics of POST Complex Formation

**Figure 5:**
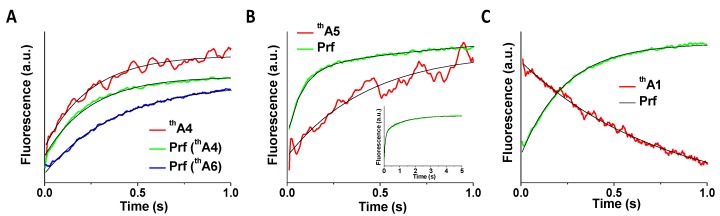
Kinetics of POST complex formation of ^th^A-containing mRNAs. Fluorescence changes on rapid mixing of PRE complexes (0.1 mM) programmed with ^th^A-containing mRNAs (black) and EF-G.GTP (2 mM) compared with fluorescence changes of fMetLys-tRNA^Lys^(prf) (red), present as part of the PRE complex. (**A**) ^th^A4 and ^th^A6; (**B**) ^th^A5; (**C**) ^th^A1. Solid black lines are exponential fits with *k*_app_ as shown in Table 3. The inset in (**B**) shows an expanded time scale.

Apparent rate constants for EF-G.GTP-dependent translocation of PRE complexes programmed with ^th^A-containing mRNAs to form POST complexes were determined from the time-dependent changes in fluorescence intensity of either ^th^A (*k*_A_) or fMet-aa-tRNA^aa^(prf) (*k*_prf_) [17,18] (Figure 5, Table 3; the lack of a spectral change precluded *k*_A_ determination for PRE complexes programmed with ^th^A6).

The relative values of *k*_A_ vs. *k*_prf_ were different for PRE complexes programmed with ^th^A4, ^th^A1, or ^th^A5. For ^th^A4-programmed ribosomes *k*_A_ is indistinguishable from *k*_prf_, indicating that the movements of tRNA and mRNA are strongly coupled. In contrast, *k*_prf_ for ^th^A1-programmed PRE complexes is 3-fold larger than *k*_A_. This most likely reflects a more dramatic change in ^th^A1 fluorescence on tRNA^fMet^ dissociation from the ribosomal E-site, a step following translocation. Movements of tRNA and mRNA appear quite decoupled for ^th^A5-programmed PRE complexes, with the apparent rate constants for the major and minor changes in prf fluorescence intensity, *k*_prf1_ and *k*_prf2_, being 6-fold larger and 2-fold smaller, respectively, than *k*_A_. The rate constant *k*_prf1_ for ^th^A5 is also considerably larger (13 s^−1^) than the values of *k*_prf_ for ^th^A1, ^th^A4, and ^th^A6 (2.6–4.4 s^−1^), an acceleration that might arise from weaker codon:anticodon interaction (see Section 3). The similarity in the values of *k*_A_ for ^th^A1 and of *k*_prf2_ for ^th^A5 (Table 2) suggests that the latter may also correspond to tRNA^fMet^ dissociation.

## 3. Discussion

The results presented here show that ^th^A, like ^th^G [24], can be incorporated into functional mRNAs, giving rise to fluorescence intensity changes on conversion of 70SICs to PRE complexes and PRE complexes to POST complexes. These changes make possible the monitoring of microenvironment changes of specific codon nucleotides during a cycle of polypeptide elongation, allowing measurement of the kinetics of both PRE complex formation on cognate TC binding to the ribosome and of mRNA translocation catalyzed by EF-G.GTP. Our most interesting results were obtained for PRE complex formation on the first AAA elongation codon, and it is on these results that we will focus in this section. The rate constants presented in Table 2 demonstrate that whereas ^th^A substitution for adenosine at the first and third positions (^th^A4 and ^th^A6) show little or no interference with normal mRNA function both in TC binding (*k*_1_, 120–170 µM^−1^·s^−1^) and in the overall rate constant for aa-tRNA accommodation following TC binding [*k*_2_*k*_3_/(*k*_2_ + *k*_3_), 6 s^−1^], major perturbations in mRNA function are observed for ^th^A substitution in the middle position (^th^A5), including a 3–4-fold decrease in the rate of TC binding (40 µM^−1^·s^−1^) and a much larger 15-fold decrease in the overall rate of aa-tRNA accommodation (0.4 s^−1^).

We attribute these ^th^A5-induced perturbations to the greater sensitivity on the middle codon position to structural changes arising from substitution of the bulkier, less polar, thiophene C-S-C grouping for the N^7^-C^8^-N^9^ purine grouping. Although we posit that TC binding (step 1, Scheme 1) does not involve codon:anticodon base pairing, the more extensive backbone-rRNA H-bonding network at the middle codon position [1,34] could make it more sensitive to structural change than at the 5′ and 3′ codon positions. This interpretation is consistent with results measuring the effects of disruption of the H-bonding network on TC binding, which are more pronounced at the 5-position than at the 4- or 6-positions [35]. The larger effect on the overall rate constant for aa-tRNA accommodation is also reflected in the large differences in the time courses of ^th^A fluorescence change that directly monitors codon:anticodon interaction, which is biphasic for ^th^A5 with rate constants of 40 s^−1^ (minor phase) and 0.4 s^−1^ (major phase) but monophasic for ^th^A4 and ^th^A6, with rate constants of ~10–11 s^−1^. These differences raise two major questions. First, why does the major fluorescence change with ^th^A5 proceed so much more slowly than the monophasic changes observed with ^th^A4 and ^th^A6? Second, why is the ^th^A5 fluorescence change biphasic?

In addressing the first question we note that codon:anticodon base pairing at the middle position is subject tighter structural constraints within the ribosomal decoding center than at the 5′ and 3′ codon positions [34,36,37], in accord with the observation that misreading by the ribosome occurs least frequently at the middle codon nucleotide [38,39]. Specifically, A5:U is stacked between two adjacent base pairs, which form a tight cavern. In contrast, neither 5′-A4:U nor 3′-A6:U, which each have only one stacking interaction, are faced with such a cavern. The “solvent volume” of a base-pair, defined as the volume within a cutoff radius (5 Å) from the base-pair that can be occupied by a solvent molecule [40], provides a quantitative measure of the steric constraint imposed by this cavern and is considerably reduced for A5:U as compared with either A4:U or A6:U (Table 4). Higher constraint at the middle base-pair position provides at least a partial rationale, based on steric crowding, for the dramatically reduced rate of base-pairing. Other factors may also be in play. Thus, PRE complex formation using mRNAs containing the guanosine surrogate ^th^G (Figure 1A), showed a much less marked reduction in the rate of ^th^G fluorescence change when ^th^G is placed at the middle base pair as compared with the 5′ and 3′ base-pairs [28], even though solvent volumes calculated for G:C base pairs also show a much reduced value for the middle base pair (Table 4). A clear rationale for the apparent context dependence of the rate reduction phenomenon is currently lacking, but might be related to the much more stable stacking and hydrogen bonding interactions generally seen for G-C as opposed to A-U base pairs [41,42].

With regard to the biphasic nature of ^th^A5 fluorescence change, we speculate that the answer lies in the detailed kinetic mechanism by which the three base pairs in the codon:anticodon helix are formed, whether simultaneously or sequentially. Although such helix formation in solution proceeds with a single apparent rate constant, it has been assumed that multiple base pairings occur in a series of rapid, unresolved steps [43], in accord with simulation studies of RNA helix formation [44,45]. The same scenario would explain why virtually all of the fluorescence change on conversion of C_1_ to PRE complex with either ^th^A4-mRNA or ^th^A6-mRNA occurs in Step 2 (Table 2). However, the putative slow formation of the middle base pair with ^th^A5-mRNA affords partial kinetic resolution of the base pairing steps. Accordingly, we interpret the rapid, minor phase for ^th^A5 as corresponding to the formation a first base pair to either A4 or A6, and the very slow, major phase to formation of the second base pair to ^th^A5, which is followed by much the more rapid formation of the third base pair. Interestingly, the 3.7 ± 1.5-fold higher *k*_2_ value for ^th^A5-mRNA vs. ^th^A4- or ^th^A6-mRNA (Table 2), which we attribute to formation of the first base pair, is the approximate value one would expect if individual base pairs to ^th^A4- or ^th^A6-mRNA are formed sequentially with each having a first-order rate constant equal to the *k*_2_ value found for ^th^A5-mRNA. Very slow formation of the base pair with ^th^A5 would make that step rate-determining for all subsequent steps leading to PRE complex formation, accounting for the similarity in the apparent rates of the major decreases in the fluorescence intensities of both ^th^A and tRNA^fMet^(prf) (Table 1).

In summary, our results indicate that emissive isosteric purine nucleoside mimetics can be used not only to measure rates of specific steps in ribosome-catalyzed polypeptide elongation, but also to probe position-dependent constraints that modulate the rate of codon:anticodon helix formation, particularly at A-U base pairs. In this connection, it would be interesting to extend these studies to the recently synthesized isothiazolo-based nucleosides, in which the CH group of ^th^A and ^th^G is replaced by N, in a position corresponding to the purines’ N7 [46]. Extension of this approach to examination of the helix formation within other RNA structures, such as ribozymes, IRESs and riboswitches, should be relatively straightforward.

## 4. Materials and Methods

GTP and puromycin were obtained from Sigma-Aldrich (St. Louis, MO, USA). *Escherichia coli* tRNA^fMet^ and tRNA^Lys^ were obtained from Chemical Block (Moscow, Russia). Previously reported procedures were used to prepare tight-coupled 70S ribosomes from *E. coli* MRE600 cells [31], cloned *E. coli* His-tagged proteins IF1, IF2, IF3 and EF-G [31], fMet-tRNA^fMet^ and Lys-tRNA^Lys^ [18]. Proflavin (prf) labeled tRNAs were prepared similarly to described procedures [18] except that tRNAs, reduced with NaBH_4_, were aminoacylated (and, in the case of fMet-tRNA^fMet^, formylated) and purified on FPLC (MonoQ) before being labeled with prf [24]. Proflavin labeling stoichiometries were 1.0 ± 0.1 prf/tRNA and 1.1 ± 0.1 prf/tRNA for fMet-tRNA^fMet^(prf), labeled at position 20, and Lys-tRNA^Lys^(prf), labeled at position 16, 17, or 20, respectively.

### 4.1. Synthesis of Modified mRNAs

mRNAs were prepared using standard solid-phase phosphoramidite synthesis on an Expedite 8909 synthesizer on a 500 Å CPG (1 μmol scale) column. Commercially available reagents and phosphoramidites (Glen Research, Sterling, VA, USA), and a ^th^A-phosphoramidite, prepared as described [25], were employed. Cleavage from the solid support and deprotection were accomplished with 50:50 mixture of MeNH_2_ in water (40 wt %) and in ethanol (33 wt %) at 35 °C for 6 h. The 2′-TOM group was removed by TEA·3HF at 65 °C for 3 h and the residue was desalted by precipitation (Glen Report 19–20, Glen Research). All oligonucleotides were purified by preparative polyacrylamide gel electrophoresis (PAGE) using the crush and soak method; the desired band was cut out, pulverized, extracted with 50 mM TEAA (pH 7.0) for a minimum of 12 h (while shaking) and decanted. The buffer containing the purified oligonucleotide was lyophilized and the residue was taken up in 0.2 M TEAB (pH 7.0) buffer and desalted on a Sep-pak C-18 (Waters, Milford, MA, USA). The oligonucleotides were eluted with 40% acetonitrile in water. All oligonucleotides were >98% pure as determined by analytical high resolution PAGE (Figure S3). The structures were confirmed by MALDI-TOF mass spectrometry (^th^A1, calculated M 9567.6, found 9588.0 [M + Na − H]^+^; ^th^A4, calculated M 9567.6, found 9586.1 [M + Na − H]^+^; ^th^A5, calculated M 9567.6, found 9591.9 [M + Na − H]^+^; ^th^A6, calculated M 9567.2, found 9589.6 [M + Na − H]^+^). Purified oligonucleotides were quantified by UV absorbance at 260 nm at 70 °C.

### 4.2. Complex Preparation and Quantification

70SIC was prepared, purified and quantified following a previously published procedure [28]. Modified mRNA was used as half of 70S concentration for experiments presented in Figure 2 or 2-fold excess over 70S for all other experiments. All experiments reported in this paper used purified 70SIC except for those shown in Figure 2B, for which the ultracentrifugation step described in [28] was omitted. TC was formed as described [28]. PRE complex was formed, purified and quantified by the amount of co-sedimenting [3H]-Lys [28]. All experiments reported in this paper used purified PRE complexes. POST complexes were prepared and quantified as described [28].

### 4.3. Fluorescence Experiments

Fluorescence experiments were carried out in Buffer A. Steady-state fluorescence was measured on a Fluorolog-3 spectrofluorometer (Horiba Jobin Yvon, Edison, NJ, USA). Emission and excitation spectra for ^th^A-containing mRNAs were determined using excitation at 360 nm and monitoring emission at 410 nm, respectively. PRE complex traces shown in Figure 3 are the average of two independently determined traces: (i) PRE complex prepared in situ by addition of TC to 70SIC; (ii) purified PRE complex used in the preparation of POST complex. To account for small concentration differences arising from the two preparation methods, the traces for purified PRE complexes were normalized to those for in situ generated PRE complexes, using an 8 nm window covering the fluorescence maximum to generate a normalization factor. The resulting two very similar traces were then averaged. POST complexes were prepared in situ from purified PRE complexes by incubation with EF-G.GTP (2.5 µM for 20 s), and traces were normalized using the same normalization factors as above. For all traces background fluorescence of the corresponding complex made with unlabeled mRNA was subtracted from the observed fluorescence of each labeled ribosome complex prior to normalization to the peak intensity value of 70SIC for each mRNA. For Figure 3E, multiple determinations of single experiments indicate that fluorescence intensity differences of less than 10% are unreliable because of small errors associated with both pipetting and background fluorescence subtraction.

Kinetic experiments were carried out at 25 °C on a KinTek SF-300X stopped-flow spectrofluorometer (KinTek, Snow Shoe, PA, USA). The excitation wavelengths and band-pass filters used were: 360 nm and 410 ± 10-nm for ^th^A-containing mRNAs; 462 nm and 515 ± 15-nm for proflavin. In each independent kinetic experiment, traces of fluorescence intensity changes were obtained as an average of at least 4 shots. In stopped-flow experiments containing both ^th^A and a prf-labeled tRNA, ^th^A and prf signals were collected on successive pulses from the same syringes. Each experiment was performed at least twice.

### 4.4. Solvent Volume Calculations

Structural models of codon-anticodon-ribosome interactions were based on structures of Yusupova and co-workers (protein databank accession code 3TVF [34]). Bases in the codon and anticodon were changed via molecular replacement using the swapna routine of chimera, which replaces base moieties. Structures were subsequently checked for clashes [47]. For each individual codon-anticodon base pair considered, an environment consisting of residues of the tRNA, mRNA and ribosome within 5.5 Å was used. To extract the solvent volume of these environments, a two-probe volume voxelator method was used, with a minor probe radius of 1 Å, major probe radius of 10 Å, and high grid resolution [40]. Chimera was used to obtain the subset of these solvent volumes within distance r_bp_ of the codon-anticodon base pair of interest, with r_bp_ = 5 Å. The volumes (in Å^3^) and areas (in Å^2^) of these solvent volumes were calculated with Chimera.

### 4.5. Apparent Rate Constants

Apparent rate constants (*k*_app_s) were obtained by either single (Equation (1)) or double (Equation (2)) exponential fitting of each independent experiment using Origin (OriginLab), respectively.


F = F_0_ + F_1_e^−*k*′^_1app_^t^ (1)

F = F_0_ + F_1_e^−*k*^_app1_^t^ + F_2_e^−*k*^_app2_^t^ (2)

## Figures and Tables

**Table 1:** Apparent rate constants (s^−1^) for reactions between 70SICs programmed with ^th^A-containing mRNAs and TC (0.4 µM) ^1^.

mRNA	*k*_TC_^2^	*k*_thA_	*k*_fMet_^3^
^th^A4	53 ± 1	9 ± 1	5.2 ± 0.6
^th^A5	18 ± 5	13 ± 3, minor 0.5 ± 0.2, major	0.4 ± 0.1
^th^A6	62 ± 11	11 ± 1	6.6 ± 0.8
fMKFR	44 ± 3	-	8.1 ± 0.3

^1^ Ranges are average ± average deviation for 2 independent determinations. All fits were to a single exponential except for *k*_thA_ of ^th^A5, which was fit to a double exponential. In the latter case, the major change in fluorescence intensity was 2.2 fold greater than that of the minor change. ^2^ Phe-TC binding to 70SIC proceeds at ~60 s^−1^ at 0.4 µM TC, 20 °C [33]. ^3^ Phe-tRNA^Phe^ TC accommodation proceeds at ~4 s^−1^ at 25 °C [31].

**Table 2:** Microscopic rate constants fit to Scheme 1 and relative ^th^A fluorescence intensity changes.

mRNA	^th^A4	^th^A6	^th^A5
**Rate Constants**			
*k*_1_ (µM^−1^·s^−1^)	123 ± 13	170 ± 9	42 ± 1
*k*_2_ (s^−1^)	10.6 ± 0.3	11.3 ± 0.1	40 ± 16
*k*_3_ (s^−1^)	12.0 ± 0.4	15.0 ± 0.5	0.44 ± 0.07
**Relative Intensity Change from 70SIC ^1^**			
C_1_	0.14	0.16	0.05
C_2_	1.00	1.00	0.35
PRE	1.00	1.00	1.00

^1^ Relative fluorescence intensity change on conversion of 70SIC to PRE complex is set equal to 1.00.

**Table 3:** Apparent rate constants for POST complex formation from PRE complexes ^1^.

mRNA	*k*_A_ (s^−1^)	*k*_prf_ (s^−1^)
^th^A4	4.4 ± 0.3	4.4 ± 0.3
^th^A6	n.d. ^2^	2.6 ± 0.2
^th^A1	1.3 ± 0.1	4.2 ± 0.2
^th^A5	2.3 ± 0.2	13 ± 1, major ^3^ 0.94 ± 0.16, minor ^3^

^1^ Ranges are ±average deviation from 2 independent determinations. ^2^ not determined. ^3^ Fluorescence change is biphasic (top, *k*_prf1_, bottom, *k*_prf2_). The major change in fluorescence intensity was 1.7 fold greater than that of the minor change.

**Table 4:** Solvent volumes of A-U and G-C base pairs ^1^ at first elongation codon.

Codon	Codon Position	Volume (Å^3^)
AAA	5′-A4	424
	middle-A5	271
	3′-A6	396
GUG	5′-G4	419
CGU	middle-G5	252
GUG	3′-G6	406

^1^ Ref. [28].

## Supplementary Materials

Supplementary materials are available online.
