# Supplementary material for: Stringent Nucleotide Recognition by the Ribosome at the Middle Codon Position
Source: Molecules. 2017 Aug 29;22(9):1427. doi: 10.3390/molecules22091427 (PMC5753802; doi:10.3390/molecules22091427)
Supplement: Supplementary file 1 [file molecules-22-01427-s001.pdf]

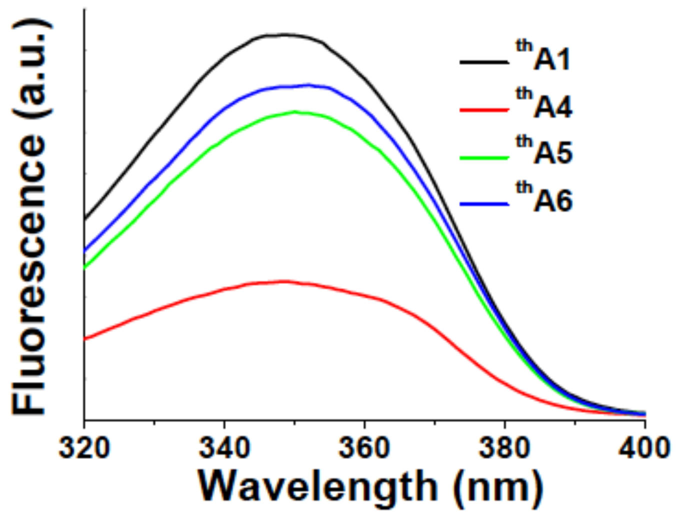

Figure S1. Excitation spectra of free thA-containing mRNAs (0.8 μM). Background fluorescence from the buffer used was subtracted. We chose 360 nm as the excitation wavelength for experiments demonstrating fluorescence changes on conversion of 70SIC to PRE complex and of PRE to POST complex (Figure 3) in order to minimize background fluorescence from the ribosome.

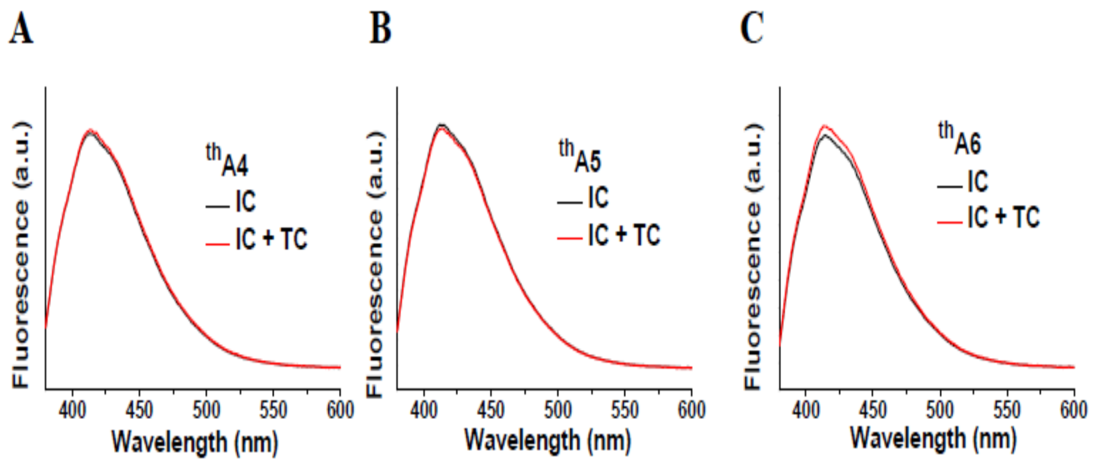

Figure S2. Lack of fluorescence change on adding non-cognate TC (EF-Tu.GTP.Arg-tRNA^{Arg}) to 70SICs programmed with thA-containing mRNAs. Traces are shown for 70SIC alone (black) and 70SIC with added non-cognate TC (red; EF-Tu.GTP.Arg-tRNA^{Arg}). For all traces, background fluorescence of the corresponding complex made with unlabeled mRNA was subtracted from the observed fluorescence of each of the labeled ribosome complexes. (**A**) thA4; (**B**) thA5; (**C**) thA6. All experiments were performed in duplicate.

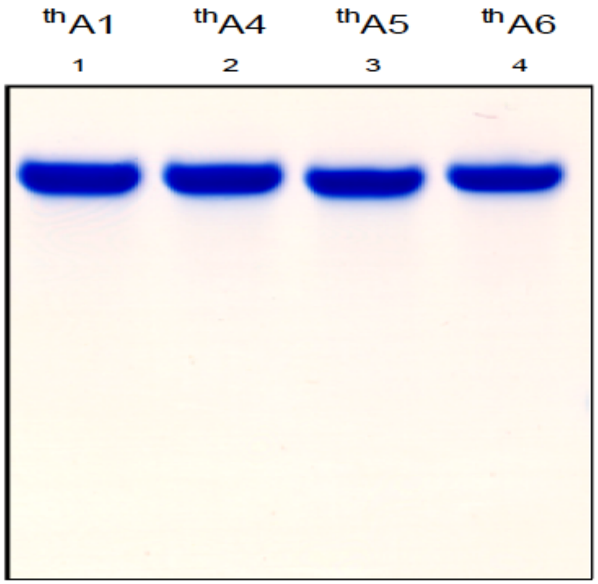

Figure S3. Analytical high resolution PAGE of oligonucleotides. Modified RNAs were analyzed by electrophoresis on a denaturing 20% polyacrylamide gel.
